# Understanding the Variation of Bacteria in Response to Summertime Oxygen Depletion in Water Column of Bohai Sea

**DOI:** 10.3389/fmicb.2022.890973

**Published:** 2022-06-09

**Authors:** Jing Wang, Xiaoxiao Guo, Yanying Li, Guisheng Song, Liang Zhao

**Affiliations:** ^1^Tianjin Key Laboratory of Animal and Plant Resistance, Tianjin Key Laboratory of Conservation and Utilization of Animal Diversity, Tianjin Normal University, Tianjin, China; ^2^School of Marine Science and Technology, Tianjin University, Tianjin, China; ^3^College of Marine and Environmental Sciences, Tianjin University of Science and Technology, Tianjin, China

**Keywords:** Bohai Sea, oxygen depletion, bacteria, 16S rRNA, *nosZ* gene, community variation

## Abstract

Aiming to reveal the variation in bacteria community under oxygen depletion formed every summer in water column of central Bohai Sea, a time-scenario sampling from June to August in 2018 at a 20-day interval along one inshore–offshore transect was settled. Water samples were collected at the surface, middle, and bottom layer and then analyzed by high-throughput sequencing targeting both 16S rRNA and *nos*Z genes. Compared to the surface and middle water, oxygen depletion occurred at bottom layer in August. In top two layers, Cyanobacteria dominated the bacterial community, whereas heterotrophic bacteria became dominant in bottom water of Bohai Sea. Based on the time scenario, distinct community separation was observed before (June and July) and after (August) oxygen depletion (*p* = 0.003). Vertically, strict stratification of *nosZ* gene was stably formed along 3 sampling layers. As a response to oxygen depletion, the diversity indices of both total bacteria (16S rRNA) and *nosZ* gene-encoded denitrification bacteria all increased, which indicated the intense potential of nitrogen lose when oxygen depleted. Dissolved oxygen (DO) was the key impacting factor on the community composition of total bacteria in June, whereas nutrients together with DO play the important roles in August for both total and denitrifying bacteria. The biotic impact was revealed further by strong correlations which showed between Cyanobacteria and heterotrophic bacteria in June from co-occurrence network analysis, which became weak in August when DO was depleted. This study discovered the variation in bacteria community in oxygen-depleted water with further effort to understand the potential role of denitrifying bacteria under oxygen depletion in Bohai Sea for the first time, which provided insights into the microbial response to the world-wide expanding oxygen depletion and their contributions in the ocean nitrogen cycling.

## Introduction

Microbes in marine ecosystem are play the important roles in regulating the biogeochemical cycle and energy transfer, which processes are commonly determined by the diversity and distribution of bacteria functional groups, such as the transformation of nitrogen in world ocean (Voss et al., 2013; Pajares and Ramos, 2019). The ecological interactions between autotrophic bacteria and heterotrophic bacteria are among critical links within global nutrient cycles (Fu et al., 2020). Typically, the productivity gradient generated by autotrophic bacteria from different locations is ascribed to community composition, which is also one of the explanations for the separation of heterotrophic bacterial communities depending on availability and quality of organic matter from primary production (Fortunato and Crump, 2011). On the other hand, remineralization and catalytic decomposition of organic substrates by heterotrophic bacteria are the keys for the nutrient availability for the primary production as well (Ward, 2013).

Nowadays, with the widespread and rapid changes in biospheric nitrogen concentrations generated by anthropogenic activity, the global impacts on biodiversity and ecosystem productivity have posed an environmental hazard in marine ecosystem (Penuelas et al., 2020; Yu et al., 2020). Therefore, understanding the nitrogen removal pathway mediated by microorganisms is of great importance for alleviating excess nitrogen (Babbin et al., 2014; Wang J. et al., 2019). Denitrification together with anaerobic ammonium oxidation (Anammox) is proved to be two major pathways to remove nitrogen under oxygen-depleted region to oxygen minimum zone (OMZ) (Ward, 2013; Babbin et al., 2014). Before the discovery of Anammox, denitrification mediated by a serial of microorganisms is estimated to account for over 50% of dissolved inorganic nitrogen removal from world ocean (Reyes et al., 2017). There are many reports on nitrogen removal by denitrifying bacteria in OMZs, such as the water column of Arabian Sea (Wyman et al., 2013), the Yangtze River Estuary, and the Pearl River Estuary of China (Wang J. et al., 2019; Li et al., 2020). Understanding the denitrification bacteria and their contribution at each specific steps is critical for estimating the nitrogen budget of the atmosphere and oceans, especially under the changing DO, since oxygen content is a regulating factor in promoting the process of denitrification. At the same time, emission of the greenhouse gas – nitrous oxide (N_2_O) is effectively alleviated in the last step of denitrification, in which the enzyme encoded by the *nosZ* gene converts N_2_O into N_2_ and releases it into the atmosphere (Zumft, 1997). Consumption of oxygen by bacteria respiration leading to the seize of nitrate (NO_3_^–^) as electron acceptor will eventually increase global warming due to the release of N_2_O (Rabalais and Turner, 2019). Among the diverse bacteria that participating in complete denitrification, *nosZ* gene is an accurate and also highly effective biomarker for evaluating denitrifying communities especially with the estimations of greenhouse gas emission in a wide range of environment (Kandeler et al., 2006; Wang J. et al., 2019).

The area of the marine hypoxic zone attributable to climate change varies with depth and emerges a notable expansion trend (Deutsch et al., 2011), resulting in typical OMZs, such as the South Pacific (Sun et al., 2017), northern tropical Indian Ocean (Menezes et al., 2018), North Atlantic (Klar et al., 2018), and Southern Ocean (Levin, 2018), where the increase of N_2_ production results in subsequent restriction of global primary production (Deutsch et al., 2011). Dissolved oxygen (DO) is a key parameter for bacteria physiology, and DO gradient has strong correlations with the structure of bacterial community (Spietz et al., 2015). High incidence of oxygen depletion increases denitrification rates, accelerates the recycling of inorganic nitrogen by improving dissimilatory nitrate reduction to ammonium (DNRA), which processes enhance the retention of both organic and inorganic nitrogen, and exacerbates oxygen deficiency further (Jäntti and Hietanen, 2012; Song et al., 2021). Moreover, transcripts of denitrifiers are also confined to the low oxygen level (Suter et al., 2020). Yet, researches on the effects of hypoxia on microbial composition are still limited in inland seas globally, including China. To figure out how individual groups of bacteria respond to decreasing DO functionally, effect of oxygen depletion on microbial communities is crucial to reveal the consequences of outspreading OMZs globally.

Bohai Sea is a semi-enclosed inland sea located in the northeastern part of China (37°07′N–40°56′N, 117°33′E–122°08′E). Due to the continuous nutrients input from the Yellow River and the Haihe River, Bohai Sea is subjected to a high eutrophication level with yearly average loading of nitrogen up to 243.2 mg/L (Peng, 2015; Qiao et al., 2017). Moreover, increasing industrialization and population in surrounding cities also significantly intensifies the degree of eutrophication during the past 60 years (Yang et al., 2011). As a consequence of excessive emission of nutrients and organic pollutants, bottom oxygen depletion occurred every summer in the central Bohai Sea (Zhai et al., 2012), and the oxygen-depleted area had expanded to 1,200 km^2^ in August of 2014 (Jiang et al., 2016), covering the Liaodong Bay and the northern part of the Bohai Sea at a greater extent (Wei et al., 2019). Although the biogeochemical cycling driven by microbes has displayed instant or delayed response to oxygen depletion (Bertics et al., 2013; Sinkko et al., 2013), less is known for the microbial communities in the reported oxygen depletion water of Bohai sea. The contribution of denitrifying bacteria in Bohai Sea has been revealed by denitrification rate, relative abundance, and NO_3_^–/^nitrite (NO_2_^–^) reductase activity of *nar*/*nir*-encoded denitrifiers in sediments (Wang et al., 2015; Zhang et al., 2019; Chen et al., 2020). However, information on the *nosZ*-type denitrifying bacteria and their metabolism capability in Bohai Sea water column is still unknown, and the potential correlation between denitrifying bacteria and the formation of the bottom water hypoxia is also awaiting to discover.

In this study, surface, middle, and bottom layer water samples were collected along an inshore–offshore transect covering the reported hypoxic zone in Bohai Sea following a time scenario under the formation of oxygen depletion from June to August of 2018. Both 16S rRNA-encoded total bacteria and *nosZ* gene-encoded denitrifying bacteria were fully investigated by high-throughput sequencing with the aim to reveal the impact of oxygen depletion on bacterial community. Based on the variations in bacteria diversity and spatial or temporal distribution patterns, in combination with local environmental parameters, effects of oxygen depletion on variations and distributions of bacteria in Bohai Sea water were evaluated. Moreover, the biotic impact between Cyanobacteria and heterotrophic bacteria was also discussed by co-occurrence network analysis. Contributions of denitrifying bacteria in nitrogen cycle under oxygen depletion in Bohai Sea water were further analyzed by coupling functional prediction by 16S rRNA gene with the distribution of *nosZ* gene in August samples.

## Materials and Methods

### Sample Collection and Physiochemical Parameter Measurement

**FIGURE 1:**
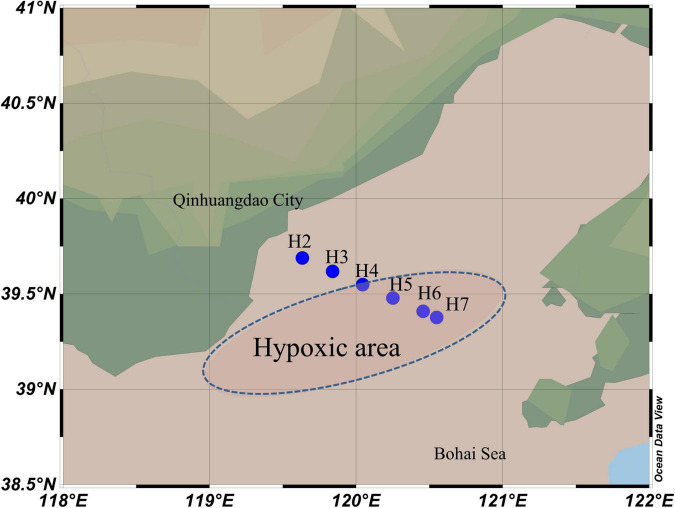
Map showing the sampling sites in Bohai Sea along the inshore–offshore transect. The reported hypoxic area was shown in the blue dash line oval.

Cruises were carried out in the Bohai Sea on 11 June, 19 July, and 8 and 26 August of 2018, respectively. A total of three layers (surface, middle, and bottom) of water samples were collected from 6 sites (H2-H7:39.38°–39.69°N, 119.63°–120.55°E) in June and 5 sites (H3-H7:39.62°–39.69°N, 119.84°–120.55°E) in the rest of 3 cruises (site H2 was occupied for aquaculture since July). One additional layer was collected at site H7 between middle and bottom layers in late August for a precise vertical profile of the DO measurement, but this sample was evaluated for geochemical parameter only. Details for the total 62 samples from 4 cruises could be found in Supplementary Table 1. The transact was settled from inshore to offshore across the southwest of the bowl-shaped depression area (Figure 1), where the oxygen depletion was reported (Zhai et al., 2012). Vertical profiles of physiochemical parameters, including temperature, salinity, depth, DO, and chlorophyll *a* (*Chla*), were acquired using an RBR maestro multi-parameters mounted to the Sea-Bird SBE-911 Plus V2 conductivity–temperature–depth (CTD) system *in situ*. Water samples were collected by 5-L Niskin bottles, and microbes were collected by filtering 1 L of water from each layer through GTTP filters (0.22 μm pore size, 47 mm in diameter, Merck Millipore, Germany) under 0.5 MPa. All the filtrations were accomplished within 0.5 h after the Niskin bottles were on board. The filters were placed into a 2-ml microtube with sterilized tweezers and then frozen in liquid nitrogen immediately. Filters were transported on dry ice to the laboratory and stored at −80°C until DNA extraction. Simultaneously, about 500 ml of each water sample was transported on ice and stored at 4°C for chemical analysis. Concentration of ammonium (NH_4_^+^), NO_3_^–^, and NO_2_^–^ was measured in the laboratory using a AA3 HR analyzer (SEAL Analytical, United States).

### DNA Extraction and Polymerase Chain Reaction Amplification

Genomic DNA was extracted and purified using lysozyme, proteinase K, and sodium dodecyl sulfate with chloroform extraction and isopropanol precipitation following Kan et al. (2006). Quality and quantity of DNA were reliably ascertained using an ND-2000 nanodrop spectrometer (Thermal Scientific, Wilmington, DE). Primers 338F (ACTCCTACGGGAGGCAGCA) and 806R (GGACTACHVGGGTWTCTAAT) with barcode attached were applied targeting the V3 and V4 regions of bacterial 16S rRNA gene (Dai et al., 2016), and amplification of *nosZ* gene was performed with the primer pairs nosZF (CGCTGTTCITCGACAGYCAG) and nosZR (ATGTGCAKIGCRT-GGCAGAA) after Rich et al. (2003).

The polymerase chain reaction (PCR) was performed in a 20 μl mixture, containing 4 μl of 5× FastPfu Buffer, 2 μl of dNTPs (2.5 mM), 0.8 μl of each primer (5 μM), 0.4 μ of FastPfu polymerase, and 10 ng of template DNA. Amplification and purification of 16S rRNA product followed the protocols in Guo et al. (2022), and protocols for *nosZ* gene were after Wang J. et al. (2019).

### High-Throughput Sequencing and Data Processing

Triplicates of the successful PCR products were mixed thoroughly, then purified, quantified, and homogenized to form a sequencing library. High-throughput sequencing was performed using Illumina MiSeq System (Illumina MiSeq, United States). The original image data files obtained by high-throughput sequencing were converted into original sequenced reads by base-calling analysis. The results were stored in FASTQ (referred to as “fq”) file format. Quality check of sequences was performed using the Quantitative Insights Into Microbial Ecology (QIIME) standard pipeline (Caporaso et al., 2010); the low-quality reads, primers, barcodes, and adaptors were trimmed off correspondingly. The operational taxonomic units (OTUs) were defined by 97% identity for taxonomy assignment by UCLUST method (Edgar, 2010). All the datasets were normalized based on the rarefaction curve, and the following analysis was made based on the flattened data. Taxonomy for each OTU was made by aligning sequences with the database from GeneBank (Release 7.3^1^).

### Statistical Analysis

Rarefaction analysis was performed using Mothur (version v.1.30) and R for all the OTUs with the aim to ensure the amount of the sequences was as reasonable as possible (Supplementary Figure 1). Mothur software was used to evaluate the species richness and alpha diversity of the samples (accumulated cyclone energy (ACE), Chao1, Simpson, and Shannon indices). Beta diversity at the class level was calculated between the 6 sites using non-metric multidimensional scaling analysis (NMDS) based on binary Jaccard distance. Bacterial community dynamics at generic level along with environmental variables were analyzed using canonical correspondence analysis (CCA) and mapping with R language vegan package. The co-occurrence network was analyzed by R (version 3.6.3), and the Pearson correlation coefficients were used to construct co-occurrence networks. The top 20 abundant OTUs were aligned with OMZ representative sequences to perform phylogenetic analysis, and the phylogenetic tree was generated using Python language tool (version 3.0). Functional potential of microbial communities in water samples was predicted using phylogenetic investigation of communities by reconstruction of unobserved states (PICRUSt2) (Langille et al., 2013).

### Nucleotide Sequence Accession Numbers

The annotated nucleotide sequences of 16S rRNA and *nosZ* genes were deposited to National Center for Biotechnology Information (NCBI) under the accession numbers SRR12403482-SRR12403543 and PRJNA815372, respectively.

## Results

### Biogeochemical Characteristics of Bohai Sea Under Oxygen Depletion

**FIGURE 2:**
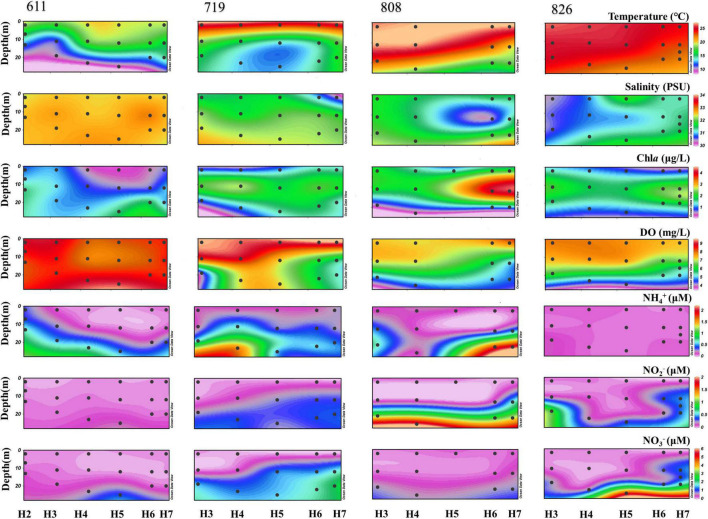
The vertical profile of biogeochemical parameters (temperature, salinity, Chl*a*, DO, NH_4_^+^, NO_3_^–^, and NO_2_^–^) of each water sample.

In this study, a clear pattern of environmental parameters was observed. Along sampling time, water temperature increased gradually from June to August, but decreased vertically as the water deepens. The salinity increased from the surface to bottom along the vertical profile, but decreased along sampling time. Other than in June, the maximum Chl*a* was detected all in middle layers, which were the depth of chlorophyll a maximum (DCM) for Bohai Sea; and the observed DO value kept decreasing vertically, with an obvious decline in bottom water ranging from 9.16 (June) to 4.21 mg/L (late August) along the time scenario. The highest and lowest DO values were all observed from the bottom water of H5 (Figure 2), which was the deepest site along the transect (Supplementary Table 1). Concentrations of NO_3_^–^, NO_2_^–^, and NH_4_^+^ kept increasing from surface to bottom, with that of the NO_3_^–^ which was always greater than NO_2_^–^ for each sample. One exception was observed in late August samples, where the concentration of NH_4_^+^ was constantly stable along the vertical profile (Figure 2).

### Diversity of Bacteria in Bohai Sea

**FIGURE 3:**
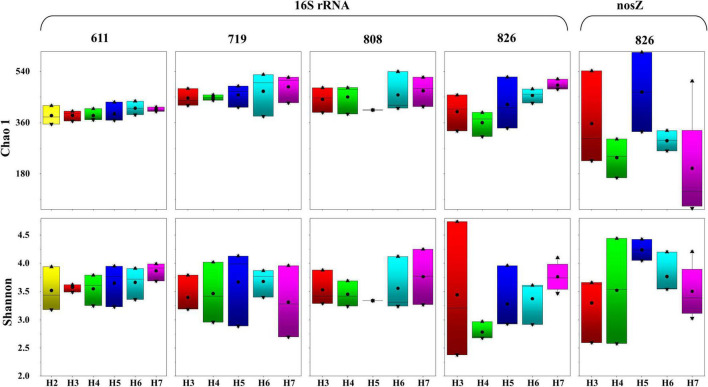
Alpha diversity of 16S rRNA and *nosZ* gene-encoded bacteria at each sampling time. Thick bar represents median, black point represents average, boxes represent the middle 50% of the data, and whiskers represent the upper and lower quartiles.

In total, 62 samples were sequenced for 16S rRNA gene with 4,919,934 reads that were obtained after sequencing, and 4,573,864 high-quality sequences were generated after paired-end splicing and filtering. After flattening at 25,000 sequences for each sample, in total, 580 OTUs were identified. A rarefaction curve was drawn to confirm that the amount of sequence was sufficient to reflect the species of the samples (Supplementary Figure 1A). The OTU numbers ranged from 299 to 488 at a cutoff value of 97% nucleotide similarity, among which, sample H7B808 contained the highest OTU numbers (448 OTUs), and the sample H4M826 contained the lowest OTU numbers (260 OTUs). Vertically, the number of OTUs identified from the bottom layer samples was greater than the surface and middle layers at all sampling time points; horizontally, the OTU numbers displayed an increasing trend from the inshore to offshore. Along the time scenario, the overall OTU numbers showed an increasing trend from June to early August and then decreased at the last sampling time point. The Chao1 and Shannon indices calculated based on the OTU numbers are summarized in Figure 3. Shannon indices ranged from 2.38 to 4.74, and Chao1 richness ranged from 311.19 to 539.47. Vertically, the alpha diversity index increased as the water layer deepens. Horizontally, the Chao1 and Shannon indices at the same water layer generally increased from inshore to offshore. Variations among sampling time scenario were displayed by increasing trend in the surface water, but peak in the middle and bottom layers for July and early August samples. Taken all the indices into consideration, samples in August displayed higher diversity, which were represented by 4.74 of Shannon in H3B826 and 539.47 of Chao 1 in H6B808.

Among the 62 samples, successful amplification for *nosZ* gene was only achieved from 15 late August samples, with 1,371,700 sequences that were obtained in total and then flattened with 10,000 sequences. A rarefaction curve was generated by the flattened data to confirm that the number of sequences was sufficient to reflect the species diversity (Supplementary Figure 1B). By aligning with the functional gene database from GeneBank (Release 7.3 see text footnote 1), 3351 OTUs were obtained after final annotation. In general, sample H5B826 contained the highest OTU numbers (425 OTUs), and sample H7M826 contained the lowest OTU numbers (50 OTUs). Vertically, the OTU numbers displayed an increasing trend from the surface to bottom layers; horizontally, no regular pattern was observed based on the OTU numbers. As displayed in Figure 3. Chao1 richness ranged from 51.50 to 536.98 and Shannon indices ranged from 2.55 to 4.43. Vertically, the Chao1 increased as the water layer deepens. Horizontally, the Chao1 and Shannon indices at the same water layer did not show a regular trend from inshore to offshore, except for samples from site H5 that displayed the highest value. In summary, with the occurrence of oxygen depletion in water, diversity of samples increased, which was represented by 4.03 of Shannon and 536.98 of Chao 1 in H5B826.

### Community Composition of 16S rRNA and *nosZ* Gene

**FIGURE 4:**
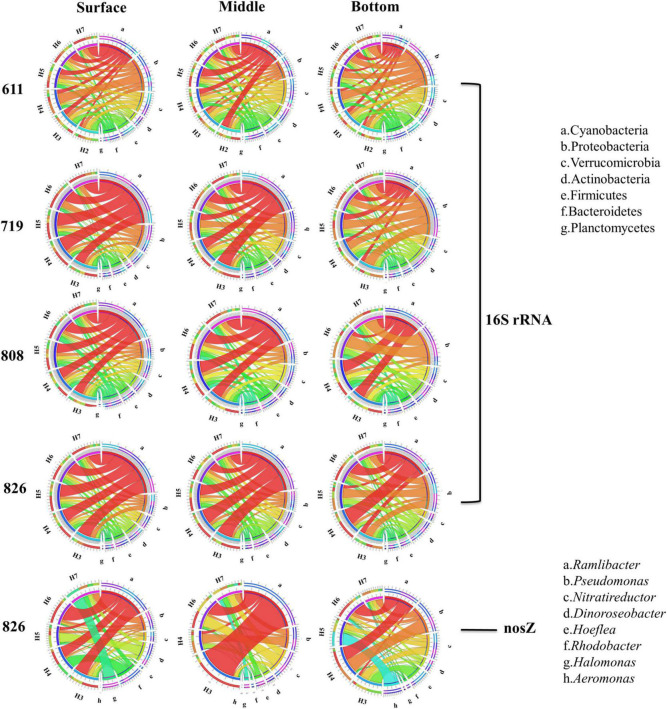
The community composition of 16S rRNA (phylum level) and *nosZ* gene denitrifiers (genus level) was revealed by pyrosequencing.

The total bacteria revealed by 16S rRNA gene at phylum level showed clear variations in the community both spatially and temporally. In Bohai Sea, the dominant phylum was Cyanobacteria, occupying 22.13% of all the community. Following was Proteobacteria (13.88%), Verrucomicrobia (7.10%), Actinobacteria (6.62%), Firmicutes (5.61%), and Bacteroidetes (5.60%). Phylum of Cyanobacteria was mainly composed of classes Oxyphotobacteria, which was the most dominant class (22%). Vertically, the relative abundance of Cyanobacteria in the surface and middle layer was more than one-third (40.60 and 38.18%, respectively), and their dominance was replaced by the Proteobacteria in the bottom water at all sites. From the top to bottom, the obvious decrease in relative abundance also included phylum Verrucomicrobia and Firmicutes, whereas the increasing trend was observed in Actinobacteria and Bacteroidetes (Figure 4). At each layer, the relative abundance of Cyanobacteria kept increasing along the time scenario, whereas the relative proportion of Proteobacteria decreased from June to August, although the absolute OTUs increased in oxygen-depleted August samples. The community composition revealed by *nosZ* gene at genus level is shown in Figure 4. The dominant genus was *Ramlibacter*, occupying 18.36% of all the detected communities, followed by *Pseudomonas* (9.71%), *Nitratireductor* (8.06%), *Dinoroseobacter* (5.51%), *Hoeflea* (4.19%), and *Rhodobacter* (3.86%). Denitrifying bacteria showed a stratification preference in each layer with site specification. Vertically, *Ramlibacter*, *Dinoroseobacter*, and *Rhodobacter* tended to distribute in surface layer whereas *Pseudomonas*, *Nitratireductor*, and *Hoeflea* were abundant in bottom water. Horizontally, *Ramlibacter* and *Rhodobacter* were abundant in coastal sites whereas *Pseudomonas* and *Nitratireductor* preferred to offshore sites. Genus *Aeromonas* replaced *Ramlibacter* and became the dominant group in sample H5B826 when the content of oxygen significantly depleted.

### Distribution of Bacterial Communities and Potential Environmental Driver

**FIGURE 5:**
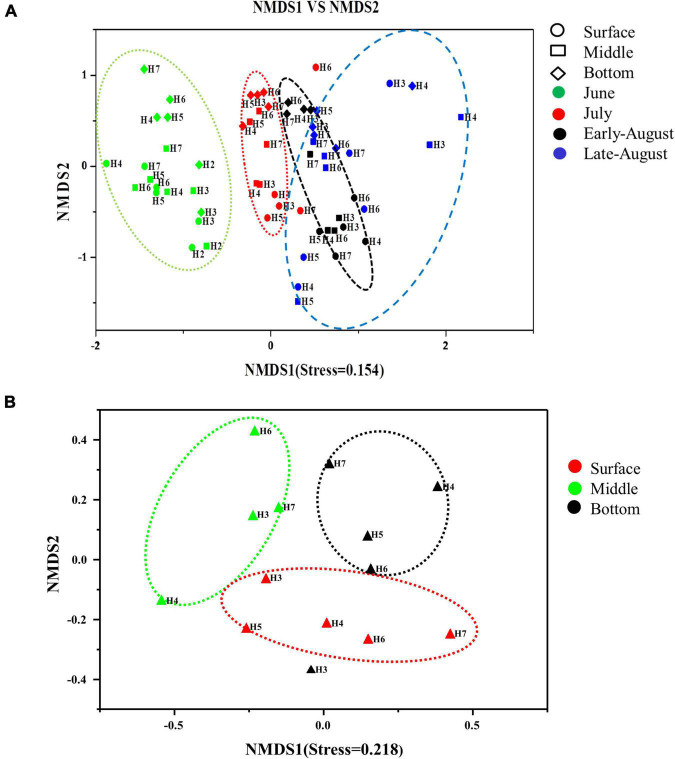
Non-metric multidimensional scaling (NMDS) plot depicting distribution pattern of 16S rRNA **(A)** and *nosZ* gene **(B)** bacteria. Axis defines 2D space that allows the best spatial representation of sample distance, based on binary Jaccard distance with stress = 0.154 **(A)**, stress = 0.218 **(B)**. The points in the figure represent different sampling times, symbols represent different water layers, and the distance between points represents the degree of difference.

Spatial (both horizontal and vertical) and temporal distributions of total and denitrifying bacteria were evaluated by non-metric multidimensional scaling (NMDS) analysis based on binary Jaccard distance. The distribution of 16S rRNA gene was well determined by sampling time scenario (stress = 0.154), with clear patterns distinguished by June, July (before oxygen depletion), and August (after oxygen depletion) (*p* = 0.046–0.003). Samples from early August and late August were grouped together, showing high similarity in community composition within the same month; samples from June were distinctly separated from the rest three sampling time points, showing the distinct community variation with sampling time (Figure 5A). Horizontally, there was no clear pattern that could be observed from each month. Vertical distribution of community was displayed by the separation of bottom samples from the surface and middle layers, but except late August. While for *nosZ* gene, vertical distribution showed a very clear pattern separated by water depth (stress = 0.218), with samples from surface, middle and bottom layers that were grouped together, respectively (Figure 5B).

**FIGURE 6:**
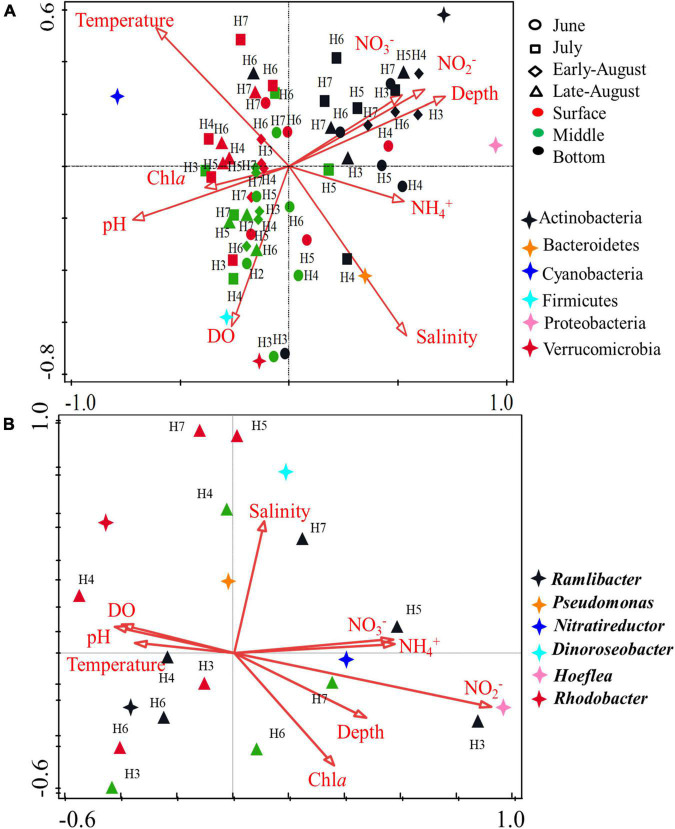
CCA ordination plot showing the relationship of environmental parameters with total bacterial (16S rRNA gene) **(A)** and denitrifying bacteria (*nosZ* gene) **(B)**. Correlations between environmental variables and CCA axes were represented by the length and angle of arrows.

Canonical correspondence analysis (CCA) was performed to spot the relationship between the environmental parameter and bacterial community. Identified phylum along with 8 environmental variables (depth, temperature, salinity, Chl*a*, NO_3_^–^, NO_2_^–^, NH_4_^+^, and DO) was analyzed (Figure 6A). The length of an environmental parameter arrow in the sorting diagram indicated the strength of the relationship of that variable with community composition. For the first two CCA dimensions, 32.93 and 44.38% of the environmental variables were explained for the total variance, and the results showed that depth, salinity, Chl*a* (*p* < 0.01), and NO_2_^–^ (*p* < 0.05) were the key impact factors shaping the bacterial community composition encoded by 16S rRNA gene. NO_3_^–^, NO_2_^–^, and depth were positively correlated with the bottom samples, indicating microbial community from bottom layer was influenced by these parameters strongly. Instead of NO_3_^–^, NO_2_^–^, and depth, bacteria communities from surface and middle layers were mostly influenced by temperature, pH, Chl*a*, and DO, among which, temperature was more positively correlated with surface samples. As for the dominant bacterial group, Actinobacteria and Proteobacteria were positively correlated with NO_3_^–^, NO_2_^–^, and depth, Bacteroidetes were highly influenced by salinity. Temperature played a key role in Cyanobacteria, especially in surface water layer. Firmicutes and Verrucomicrobia were positively correlated with DO. The results suggested that the distribution of microbial community was affected by multiple environmental factors rather than a single parameter. In addition, correlations of environmental variables and August samples revealed that DO (*p* = 0.002) was the key impacting factor shaping the community composition in oxygen-depleted water (Supplementary Table 2). The CCA analysis for *nosZ*-encoded denitrifying bacteria further confirmed the key role of DO (*p* = 0.022) on the community composition in August. Moreover, pH (*p* = 0.048) and NO_2_^–^ (*p* = 0.038) were also the impacting factors on the denitrifying bacterial community (Figure 6B). The correlations between the physicochemical factors and denitrifying bacteria in genus level showed that *Pseudomonas* and *Rhodobacter* were positively correlated with DO and pH. *Nitratireductor* was positively correlated with NO_3_^–^ content whereas *Hoeflea* was positively correlated with concentration of NO_2_^–^.

### The Network Analysis of Total Bacteria Community

**FIGURE 7:**
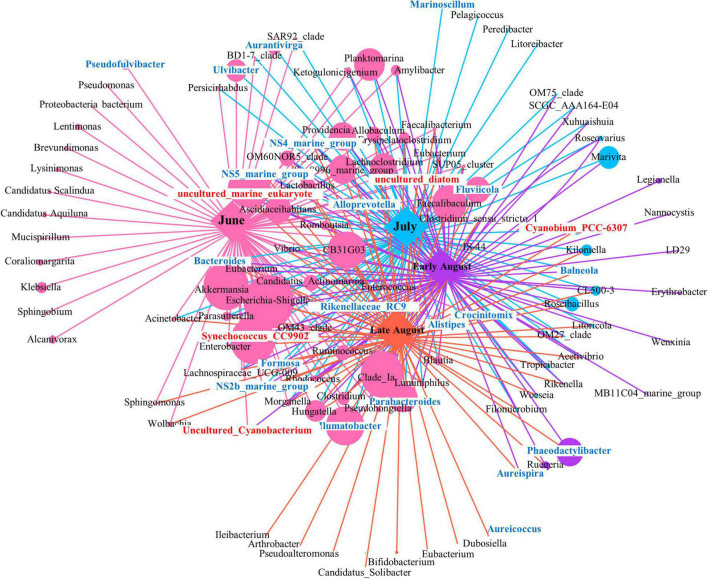
The network co-occurrence shows correlations between phototrophic and heterotrophic bacteria along the succession of sampling time. Genus of Cyanobacteria was presented in red front, and genus of Bacteroides was presented in blue front.

The co-occurrence network for 16S rRNA in genus level over different sampling times was summarized in Figure 7. Following this, the topological properties of the obtained networks were calculated to distinguish the differences in bacterial correlations. The results showed that the total bacteria in June formed highly connected and more complex networks than the other three sampling time points. The total number of nodes (bacteria identified at the genus level) was 75 for June, 20 for July, 9 for early August, and 8 for late August. Also, the abundance of bacteria (indicated by node size) had a greater value in the network within June sample (584,401). With the passage of sampling time, the number of connections represented by links (edges) first increased and then decreased in late August, and the total links were of 75 for June, 78 for July, 80 for Early August, and 75 for late August. These results indicated that with the procession of oxygen depletion, the co-occurrences between bacterial species became weak gradually. In addition, the group of *Synechococcus*_CC9902 with relatively higher abundance in June samples had a significant difference compared with other sampling times (*p* < 0.01), indicating their unique role under oxygenated environment, which was not strong anymore when DO was depleted. A significant difference was also displayed by Cyanobium_PCC-6307 (*p* = 0.004) in July and August, the same as Akkermansia (*p* = 0.014) between June and July. The connections of these autotrophic with heterotrophic bacteria showed biotic impact on community variation under oxygen depletion.

### Predicted Functional Profiling of Seawater Microbiomes

**FIGURE 8:**
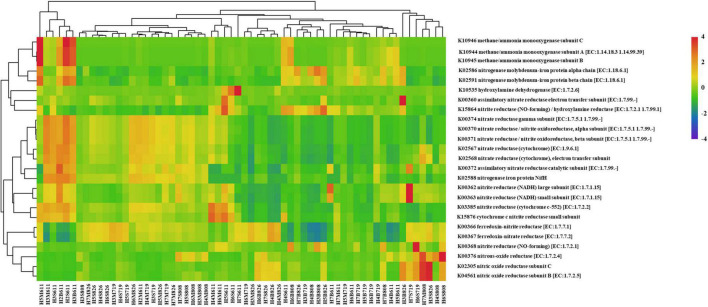
Heatmap profile showing the nitrogen metabolism-related KO based on predicted gene copy in each sample (columns represent the 25 KEGG orthology (KO) functions, rows represent the samples, and the color intensity in the heatmap represents the abundance of the functional genes).

To gain insight into the peculiar functional variations in the microbiota in water column with oxygen deficiency, corresponding metagenomes were inferred from the bacteria profiles using PICRUSt2 to show Kyoto Encyclopedia of Genes and Genomes (KEGG) pathways. A differential abundance analysis was carried out and resulting in 397 MetaCyc pathways. The clustering analysis indicated a distinguished functional profile, characterized by the enrichment in pathways involved in aerobic respiration I (cytochrome c), amino acid biosynthesis [i.e., L-isoleucine biosynthesis II and L-isoleucine biosynthesis I (from threonine)], and nitrogen cycling (i.e., urea cycle, NO_3_^–^ reduction I (denitrification), and nitrifier denitrification). Further investigation of nitrogen metabolism revealed 26 KOs (KEGG orthology) with the predicted gene copy number in the samples. Even if a certain level of dispersions was maintained, samples showed an overall tendency toward the segregation between water layers, sampling time, and sampling sites. Samples were then clustered according to the abundance profile of the 94 over-abundant pathways (Figure 8). Moreover, correlation between KOs related to denitrification and *nosZ* gene abundance was analyzed to reveal the relationship between functional prediction results and distribution of denitrifying bacteria. The results showed that KOs related to denitrification and *nosZ* gene abundance were correlated positively (*p* < 0.05) (Supplementary Table 3).

## Discussion

### Oxygen Depletion in Bohai Sea

Ever since the discovery of periodical occurrence of oxygen depletion in Bohai Sea (Zhai et al., 2012), oxygen-depleted area in Bohai Sea has been widely revealed (Zhao et al., 2017; Zhang et al., 2019). In our study, the variation in DO concentration among the 4 sampling time points is in consistent with previous observations that the intense lower oxygen concentration occurred in August bottom water (Zhai et al., 2012; Liu et al., 2019). The highly stratified and weakly hydrodynamic water column in coastal regions is confirmed to be the primary contributor to the formation of hypoxia (Hamidi et al., 2015; Irby et al., 2016; Wei et al., 2019), which is also proved to be the causes of oxygen depletion in Bohai Sea bottom waters (Zhao et al., 2017; Song et al., 2020). Although the passing of typhoon Yagi (11–16 August 2018) and Rumbia (17–21 August 2018) through the studied area has disturbed the stratification, DO in August bottom water still reaches the lowest value at station H5 (Figure 2), which is the deepest site along the transaction. In summer, disturbance also occurs when the southeast wind prevailed, under which the Yellow Sea enters and merges with the water from the central Bohai Sea and then flows out from the southern Bohai Strait (Zhang et al., 2016). Biological production, atmospheric deposition, water mixing, and release from deep sediment together with terrestrial input are all governing the source of NO_3_^–^ availability and accounting for a high concentration of NO_3_^–^ from inshore waters (Liu et al., 2003). Moreover, transportation or accumulation of nutrients with the help of bacteria decomposition guarantees the concentration when there is a lack of nutrients in seawater (Chu et al., 2010; Amin et al., 2015), which is further in support of growth for Cyanobacteria (Guerrini et al., 1998). Low dissolved inorganic phosphorus (DIP) values at oxygen depletion layer are assumed to make the environment not conducive to biological processes (Noffke et al., 2012). Such condition of low DIP accompanied with low DO is also observed at station H5. Kang et al. (2018) indicated that DIP concentration in the aerobic water column could be two times higher than anoxic environment. Additionally, high NH_4_^+^ discharge from Yellow River increases the N/P ratio in Bohai Sea, which is a noteworthy factor to shape the phytoplankton as well as the microbial compositions (Yu et al., 2018; Xin et al., 2019).

### Variation of Bacteria in Response to Oxygen Depletion in Bohai Sea

**FIGURE 9:**
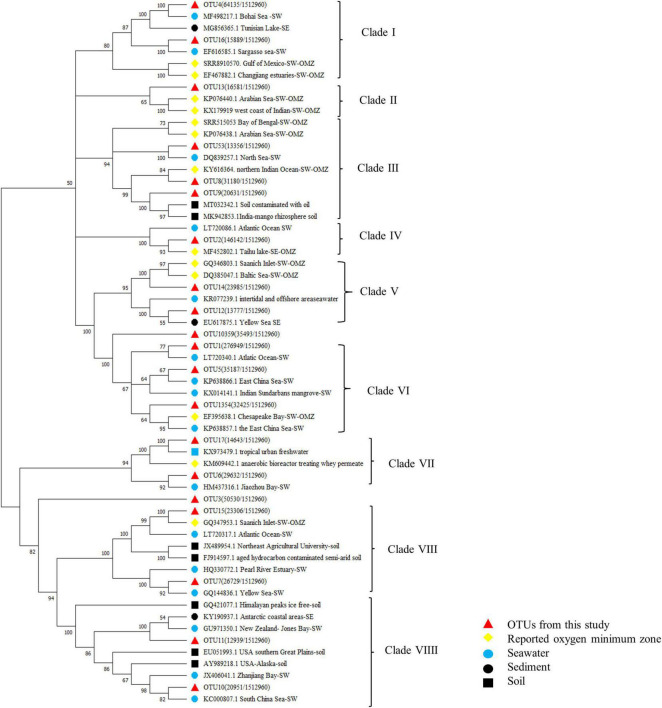
Neighbor-joining phylogenetic tree of the dominant (Top 20 OTUs) OTUs and the reference sequences from typical OMZs. Bootstrap values were 1000 replicates.

To figure out the distribution of bacterial community with the local impact of Bohai Sea, published studies from different locations of Bohai Sea revealed by high-throughput sequencing analysis are summarized in Table 1 and Supplementary Figure 3. Among the 4 studies originated from 2 geographical locations, in total, 3 transections from the coastal of Qinhuangdao city and Tianjin city to the central Bohai Sea are included. It is found that the community composition and dominant species of total bacteria differentiated based on the locations. The two studies from coastal area near Qinhuangdao city reported that the dominant groups were Proteobacteria and Cyanobacteria (He et al., 2017; Wang S. et al., 2019), which is similar to the community compositions in this study. The other two studies with locations close to Tianjin city (39.13N, 117.2E) claimed that the dominant group is heterotrophic bacteria solely, even in the surface layer water (Fei et al., 2011; Zhao et al., 2020). Location-based dominance of bacteria species has also been reported by the studies in Pacific Ocean (Li et al., 2018), Atlantic Sea (Guerrero-Feijóo et al., 2017), and Arctic Sea (Rapp et al., 2018) (Figure 9). It worth to mention that DO is always a key environmental factor in shaping the composition of bacteria in marine water (Aldunate et al., 2018; De la Iglesia et al., 2020). Concentration of Chl*a* (*p* = 0.02 in our study) is another key factor in He et al. (2017) and our study (Supplementary Table 2). Although Proteobacteria have been widely reported to be dominated in Bohai Sea (Table 1 and Supplementary Figure 3), the predominance of Cyanobacteria in this study is assumed to be attributed to both abiotic environmental factors (Figure 6) and biotic impacts (Figure 7). Bacterial distributions are susceptible to multiple physiochemical parameters, whereas spatial variability of microbial communities is argued to be subjected to nutrient availability, such as microbial available C (Weitzman et al., 2014), which is fixed by Cyanobacteria. In return, heterotrophic bacteria also promote the primary production, such as denitrification and N_2_ fixation. The microbial interactions that could impact the bacterial communities have been confirmed in the stream sediments before (Sienkiewicz et al., 2020). In our study, Cyanobacteria represented by *Synechococcus*_CC9902 and *Cyanobium*_PCC-6307 indeed are closely positively associated with heterotrophic bacteria. On the one hand, extracellular polysaccharides derived from Cyanobacteria can provide a consistent but highly diverse class of molecules to be used exclusively as the substrates for metabolic processes by heterotrophic bacteria (Amin et al., 2015; Smriga et al., 2016); on the other hand, the carbohydrate-active enzyme can catalyze the degradation of algal polysaccharides to achieve this utilization mechanism (Lombard et al., 2014), with high levels that have been detected in Bacteroidetes (Teeling et al., 2016). This assumption is supported not only by the close correlation between Cyanobacteria (represented by red font) and Bacteroides (represented by blue fonts) in network analysis, but also from the abundance variation in Bacteroides after the Cyanobacteria bloom in species composition diagram (Figure 4).

**TABLE 1:** Summary of studies on bacterial diversity and distribution in Bohai Sea.

Stations	Latitude N	longitude E	Sampling time	Sampling depth (m)	Environmental parameters							Impacting factors	Dominant phylum (top 5)	References
					DO (mg/L)	pH	Temp (°C)	Sal (‰)	NO_2_^–^ (μM)	NO_3_^–^ (μM)	NH_4_^+^ (μM)			
TJ01–TJ28	39.14°–38.63°	118.88°–117.64°	2014/08/17–19	0.5	5.01–8.32	7.92–8.27	23.5–29.4	23.5–29.7	0.13–4.39	1.66–8.61	0.33–12.78	Joint effects of environmental factors	Alphaproteobacteria Gammaproteobacteria Bacteroidetes Actinobacteria Deltaproteobacteria	Zhao et al., 2020
02, 04, 08	38.81°–38.89°	117.72°–117.96°	2006/04/12 2006/07/07 2006/10/23 2007/01/07	5.5 8.5 13.5	−	−	3.5–28.2	30.5–32.3	−	−	−	Temperature, salinity	Proteobacteria Flavobacteria Bacteroides Verrucomicrobia Firmicutes	Fei et al., 2011
S1–S5	39.36°–39.43°	119.31°–119.45°	2016/05/12	0.5 3 5	5.85–9.18	7.99–8.16	12.7–15.1	24.9–26.7	0.06–0.16	0.09–0.59	0.08–1.41	Phosphate	Gammaproteobacteria Alphaproteobacteria Bacteroidetes Verrucomicrobia Cyanobacteria	Wang S. et al., 2019
W1–W6	39.69°–39.90°	119.42°–119.74°	2014/03 2014/07 2014/10 2014/12	1	−	∼11.6	21–25	∼29	0.52–0.76	5.34–7.02	2.28–5.78	Temperature, DO, Chl*a*	Proteobacteria Cyanobacteria Bacteroidetes Actinobacteria Firmicutes	He et al., 2017
H2–H7	39.38°–39.69°	119.63°–120.55°	2018/06/11 2018/07/19 2018/08/08 2018/08/26	2 11–12 19–25	4.21–9.16	7.77–8.27	9.4–27.0	30.08–33.03	0.01–1.98	0.02–5.62	0.01–2.02	Depth, salinity, Chl*a*, NO_2_^–^, pH	Cyanobacteria Alphaproteobacteria Gammaproteobacteria Deltaproteobacteria Verrucomicrobia	This study

In this study, Cyanobacteria dominated at the top two layers before and after oxygen depletion, with a higher preference in coastal sties. Singh and Bhadury (2018) have reported the greater fraction of Cyanobacteria among bacterial composition in summer and higher relative abundances in the coastal ocean than open sea, which is in highly agreement with our results. Nutrient enrichment originated from anthropogenic sources (urban, agricultural, and industrial) as well as water discharge and atmospheric deposition has inspired Cyanobacteria expansion and perseveration (Paerl and Scott, 2010). The significant effect of NO_3_^–^ and NO_2_^–^ on the distribution of Cyanobacteria (Supplementary Table 4) in our study is further in consistent with the above conclusions. In addition, persistent vertical stratification, high water temperature (exceeding 20°C) and low flushing rates are all characterized by our study sites and also contribute to the formation and expansion of Cyanobacteria in nutrient-enriched water bodies (Paerl et al., 2001). Among the members of Cyanobacteria, *Synechococcus* lineage is most abundant in all studied sites. Positive correlation of *Synechococcus* lineage with NH_4_^+^, NO_3_^–^, and NO_2_^–^ concentrations reflects their preference to nutrients (Zwirglmaier et al., 2008). In our study, NO_2_^–^ concentration is more related to the *Synechococcus* than NO_3_^–^ (Figure 6). NO_3_^–^ uptake might be inhibited by NH_4_^+^ when concentrations >1 μmol L^–1^ (Lindell and Post, 2001). However, *Synechococcus sp*. strain CC9902 is capable of assimilating NO_2_^–^ with the presence of high NH_4_^+^ concentration (Wyman and Bird, 2007). The high concentrations of NH_4_^+^ (up to 2.34 μmol L^–1^) in most sites may inhibit the uptake of NO_3_^–^ (Supplementary Table 4). Vertically, the close correlation of light and photosynthesis may well explain the dominance of Cyanobacteria in surface layer waters. Surprisingly, the bottom water of oxygen-depleted stie (H4 and H6 in late August) is also dominated by Cyanobacteria (Figure 4), which might due to their ability for photosynthesis in the hypoxic zone (Liu et al., 2015). In addition, the vertical mixing caused by typhoons is another assumption for this unexpected distribution, since the community compositions are highly similar between surface and bottom water at these two sites. In addition, *Cyanobium*-PCC-6307 with a relative abundance of 70.29% is identified in the surface water compared to other water layers. The O_2_ depleted microzones caused by these cyanobacteria colonies may provide unexpected advantages for reducing nitrogen oxides in surface waters (Wyman et al., 2013).

Intense variations in bacteria community under the impact of oxygen depletion can be clearly observed in Proteobacteria, which are functionally related to sulfur, methane, hydroxide, sulfate reduction, and denitrification (Saarenheimo et al., 2015; Jing et al., 2020; Zhou et al., 2020). In this study, relative abundance of Proteobacteria is extremely high in bottom waters, and their dominance increases with the depletion of DO. In bottom water, Rhodobacterales are dominated within the most abundant class of Alphaproteobacteria and are highly correlated with organic particles in marine environment (Dang and Lovell, 2016). Rhodobacterales are active in the pollutant degradation when oxygen is depleted (Loy et al., 2005). SAR11, taxon followed by Rhodobacterales in Alphaproteobacteria has been reported to be ubiquitously dominated in marine surface waters (Kraemer et al., 2020). Their abundant distribution in the bottom layers in this study is most probably due to the intrusions of oligotrophic water from the eastern Bohai Sea (confirmed by CCA with a high correlation with ammonia) (Figure 6). Another possible reason is because SAR11 bacteria can catalyze the NO_2_^–^ producing step of denitrification with *nar* gene-encoding proteins, which is vital in NO_3_^–^ reduction in the anoxic zone (Tsementzi et al., 2016). Other than SAR11, the high dependence on the availability of dissolved organic matter by the abundant lineages of SAR86 and Rhodospirillaceae should be the result of accumulated NO_3_^–^ and NO_2_^–^ in the bottom waters when DO began depleted (Freixa et al., 2016). Gammaproteobacteria is the second large class of Proteobacteria in bottom water but overwhelming in top waters, which has been proven to be an adaptive group in some typical hypoxic environments, such as the northern Gulf of Mexico (Devereux et al., 2015), Black Sea (Jessen et al., 2017), and Baltic Sea (Broman et al., 2017). Additionally, SAR406 clade, which is reported particularly abundant in OMZs, also has the ability to reduce NO_3_^–^, or possibly to oxidize sulfur compounds (Thrash et al., 2017).

Variation after Cyanobacteria blooms has been found in other heterotrophic bacteria. The Eutrophication characteristics and low freshwater input in June are correlated with the high relatively abundance of Bacteroidetes in Bohai Sea, as higher nutrient levels favor their growth (Alonso-Gutiérrez et al., 2009). Bacteroidetes, aerobic bacteria, play an important role in organic matter degradation in eutrophic marine environment, which has been well confirmed by recent genome sequencing (Kabisch et al., 2014). Díez-Vives et al. (2019) describe the distribution of Bacteroidetes within the marine associated NS5 and NS2b clades that are significantly associated with depth. This is in consistent with the relative abundances of NS2b (relative abundance from the surface layer to the bottom layer is 1.5, 2.1, and 7.3%), and NS5 clades (relative from the surface layer to the bottom layer are 3.8, 6.9, and 42.2%) that high in the bottom waters in all sites. Moreover, Bacteroidetes have also been found to be associated with phytoplankton (Arnosti, 2011), but this correlation is weakened with the depletion of oxygen as shown in late August samples (Figure 7). Members of Flavobacteria have been reported to have preference with algal organic matter in the surface water partially in help to explain their distribution in studied area (Williams et al., 2013). Additionally, Flavobacterium is able to grow on some algal cells and utilize phytoplankton-derived polysaccharides because of its gliding motion characteristics (Tang et al., 2017). As for Verrucomicrobia, Freitas et al. (2012) find that fraction of Verrucomicrobia is higher in coastal and shallow water, as observed in this study. Together with Planctomycetes, Verrucomicrobia can form the part of a taxonomic super-phylum called PVC and occupying a higher relative abundance in upper waters (Spring et al., 2016). Such distribution of Verrucomicrobia has high possibility relating to their dependence on organic matter and contributing to the carbon cycle (Canfora et al., 2014), but rare has been found in oxygen-depleted deeper water.

Actinobacteria are abundant in coastal and deeper waters especially in August. It is speculated that rainfall and corresponding freshwater input from terrestrial might impact the presence of Actinobacteria since the Actinomycetes are homologous to many soil-originated bacteria from phylogenetic tree (Figure 8). Higher abundance of Firmicutes appears mostly in the samples from June, followed by late August. Sun et al. (2017) state that Firmicutes may exploit the denitrification, DNRA, or both the two pathways to remove excess NO_3_^–^ from the environment. Water layers with higher Firmicutes proportion and accordingly low NO_2_^–^ concentration in partial help to understand this distribution.

### Nitrogen Removal Under Oxygen Depletion

Nitrate reducing bacteria that utilize and transform NO_3_^–^ to N_2_O play the important roles in the nitrogen cycling in spatial variated (both horizontally and vertically) regions where DO reach hypoxic level (Gomes et al., 2020). In our study, diverse and high percentage of NO_3_^–^ reducing bacteria, such as *Nitratireductor*, *Pseudomonas*, and *Halomonas*, are detected in oxygen depletion sites, which potentially provides sufficient substrate for the N_2_O reductase encoded by the *nosZ* gene in the reported oxygen depletion area in Bohai Sea. Furthermore, significantly negative correlation of *nosZ* gene out number with DO in August confirms that oxygen depletion can increase the abundance of N_2_O consumption bacteria. This is also in consistent with the conventional conclusion that the process of N_2_O to N_2_ is the least oxygen tolerant anaerobic step in denitrification pathway (Zumft, 1997). N_2_O consuming bacteria may be vital in understanding the N_2_O flux in OMZ (Arevalo-Martínez et al., 2015). Correlation between N_2_O consumption or N_2_ generation rate and the abundance of *nosZ* gene showed that in addition to the interference of organic matter or other heterotrophic microorganisms in the environment, the abundance of functional genes was a key factor reflecting the production rate, which indicates the encoded corresponding enzymes that participate and catalyze biochemical processes (Choi et al., 2016; Zhao et al., 2017). In our study, the higher abundance of *nosZ* gene in the oxygen-depleted waters with the ability to convert N_2_O into N_2_ may help to alleviate the accumulation of the greenhouse gas N_2_O from Bohai Sea. Based on the correlations of nitrogen transformation predicted by KEGG pathways from 16S rRNA gene with the relative abundance of *nosZ* gene in this study, the pathways with positive correlations are picked up and successfully sorted back to the corresponding *nosZ* gene-encoded bacteria (Supplementary Figure 4) to confirm the processes of denitrification by bacteria, and the key impacting factor on the double confirmed groups of denitrifying bacteria is also revealed to be DO and NO_2_^–^. However, in the nearshore sites and the upper water, where the DO content is high, a considerable *nosZ* community has also been identified. The potential active *nosZ* community in these regions might capture N_2_O produced in deeper seawater and thus reduce the flux into the atmosphere (Sun et al., 2017).

*Ramlibacter* is the most abundant denitrification group in all samples, with the preference for surface water. However, there is no *Ramlibacter* identified from surface water at site H7, and *Halomonas* replaced *Ramlibacter* as the dominant group. High salinity in surface water of H5 and H7 possibly provides a preferable habitat for *Halomonas* due to their salt-tolerant properties as one of the halophilic bacteria (Vreeland et al., 1980). Members of *Halomonas* mainly use O_2_ or NO_3_^–^ as an electron acceptor, which process competes for the substrate of denitrification process by *Ramlibacter*. *Pseudomonas* is classified as an aerobic denitrifier in the presence of NO_3_^–^ under oxygen limited conditions (Lalucat et al., 2006), which is supposed to be dominant in the reported oxygen depletion zone (Zhai et al., 2012) at our sites H5-H7. Although typhoon disturbance increases the DO level, the high relative abundance of *Pseudomonas* at the bottom water of site H5 just verifies this assumption. *Nitratireductor*, a known denitrifying bacterium under the order *Rhizobiales*, has been identified to be the primary sink for fixed nitrogen in the OMZ of Arabian Sea (Bulow et al., 2010). *Nitratireductor* mainly participates in converting NO_3_^–^ into NO_2_^–^, which is a key process for N loss. Since oxygen removal will enhance the accumulation of NO_2_^–^ in the OMZ of Bay of Bengal (Bristow et al., 2017), high abundance of *Nitratireductor* (especially in oxygen-depleted bottom water column) and low content of DO together make a significant contribution to the release of N_2_ in oxygen-depleted water of Bohai Sea.

## Conclusion

In Bohai Sea water, Cyanobacteria dominated the bacteria community in the top two layers, whereas heterotrophic bacteria became dominant in the bottom water. Distinct distribution pattern of bacterial community was displayed before (June, July) and after (August) oxygen depletion. Vertically, denitrification bacteria showed a stable stratification when the oxygen depleted in bottom water of Bohai Sea. Distinct variations in specific total bacteria as well as denitrifying bacteria were found as a response to oxygen depletion. Other than location, depth, salinity, Chl*a*, NO_2_^–^, and pH together played the important roles in shaping the bacterial community. In August, DO was a key factor that determines not only the total bacteria community, but also the denitrifying bacteria composition in the studied area. In addition to environmental parameters, the interaction between microbes also affected the composition of bacteria. Furthermore, our study supplied the evidence on potential contribution of denitrifying bacteria in support of active emission of N_2_ as indicated by relative abundances of N_2_O reductase genes in Bohai Sea oxygen-depleted water.

## Data Availability Statement

The datasets presented in this study can be found in online repositories. The names of the repository/repositories and accession number(s) can be found in the article/Supplementary Material.

## Author Contributions

JW contributed to the conceptualization. XG and YL contributed to the experimental operation and writing – original draft preparation. JW and GS contributed to the writing – review and editing, and funding acquisition. GS and LZ contributed to the field sampling. All authors read and approved the final manuscript.

## Conflict of Interest

The authors declare that the research was conducted in the absence of any commercial or financial relationships that could be construed as a potential conflict of interest.

## Publisher’s Note

All claims expressed in this article are solely those of the authors and do not necessarily represent those of their affiliated organizations, or those of the publisher, the editors and the reviewers. Any product that may be evaluated in this article, or claim that may be made by its manufacturer, is not guaranteed or endorsed by the publisher.

## Footnotes

1
http://fungene.cme.msu.edu/
