# Supplementary material for: Understanding the Variation of Bacteria in Response to Summertime Oxygen Depletion in Water Column of Bohai Sea
Source: Front Microbiol. 2022 Jun 9;13:890973. doi: 10.3389/fmicb.2022.890973 (PMC9221365; doi:10.3389/fmicb.2022.890973)
Supplement: Supplementary file 1 [file Data_Sheet_1.docx]

Understanding the Variation of Bacteria in Response to Summertime Oxygen Depletion in Water Column of Bohai Sea

**Jing Wang^1*^, Xiaoxiao Guo^1^, Yanying Li^1^, Guisheng Song^2^, Liang Zhao^3^**

^1^Tianjin Key Laboratory of Animal and Plant Resistance, Tianjin Key Laboratory of Conservation and Utilization of Animal Diversity, Tianjin Normal University, Tianjin 300387, P.R. China

^2^School of Marine Science and Technology, Tianjin University, Tianjin 300072, China

^3^College of Marine and Environmental Sciences, Tianjin University of Science and Technology, Tianjin 300457, China

**^*^Correspondence:**

Corresponding author

[jwang_hku@163.com](mailto:jwang_hku@163.com)

**Funding:**

This study was supported by National Natural Science Foundation of China (52070143, 42076033, 41506182); Natural Science Foundation of Tianjin City (5KK19004).

**Acknowledgements:**

We thank the crews for their help and cooperation during the cruises.

**Supplementary materials**

**Figure legends**

**Fig. S1** Rarefaction curves depicting the effect of 16S rRNA gene (A) and nosZ gene (B) sequences numbers on the number of operational taxonomic units (OTUs) (3% distance) identified from the 62 water samplesand Late-August water samples.

**Fig. S2** CCA ordination plot showing the relationship between bacterial community and environmental parameters in August from surface, middle and bottom layers. Correlations between environmental variables and CCA axes were represented by the length and angle of arrows.

**Fig. S3** Map showing a profile of sampling stations from published studies and this study based on pyrosequencing data of bacterial 16S rRNA gene revealed from water column of Bohai Sea. The reported hypoxic area was shown in shaded circle on the map.

**Fig. S4** Network analysis of *nosZ*-encoded denitrifying bacteria and Kos related to denitrification.

**Fig. S5** CCA ordination plot showing the relationship of environmental parameters with *nosZ*-encoded denitrifying bacteria (genus level).

**
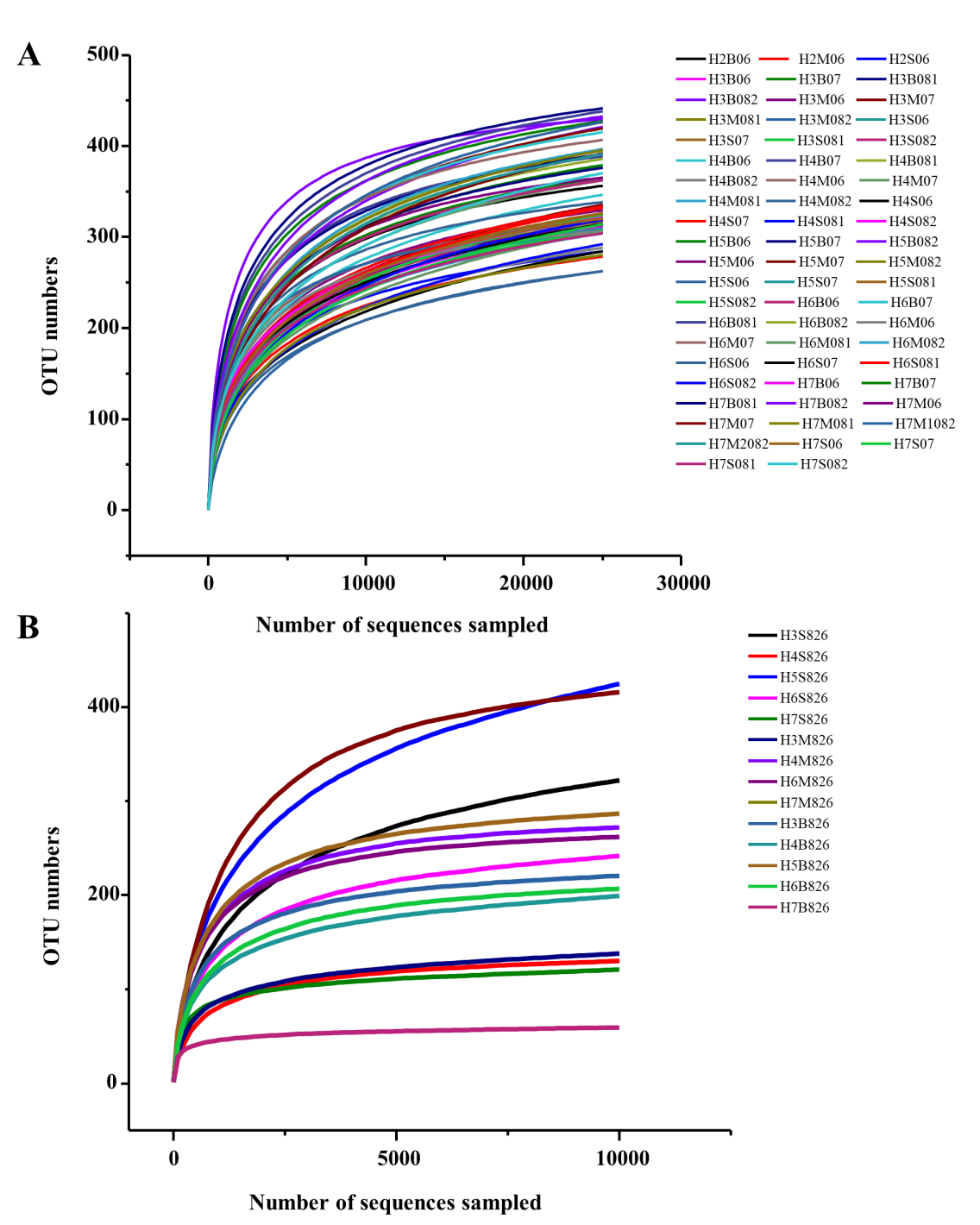
**

**Fig. S1**

**
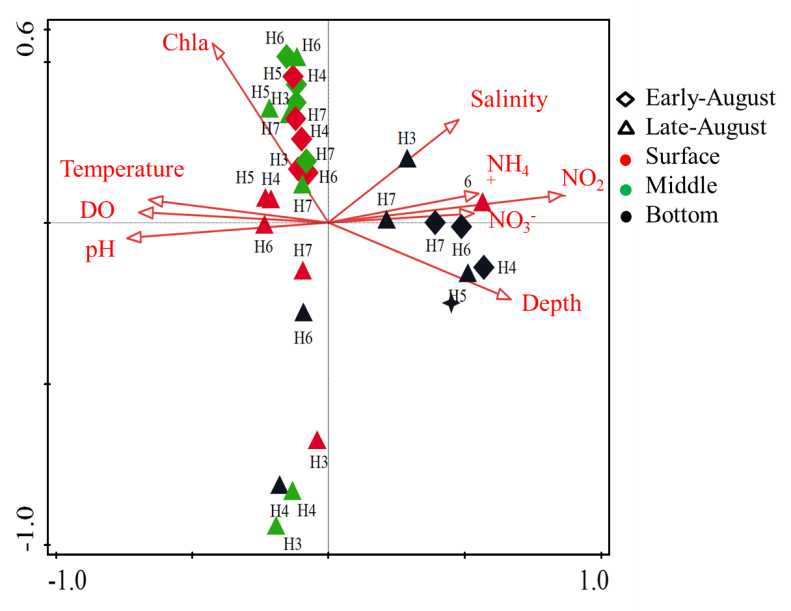
**

**Fig. S2**

**
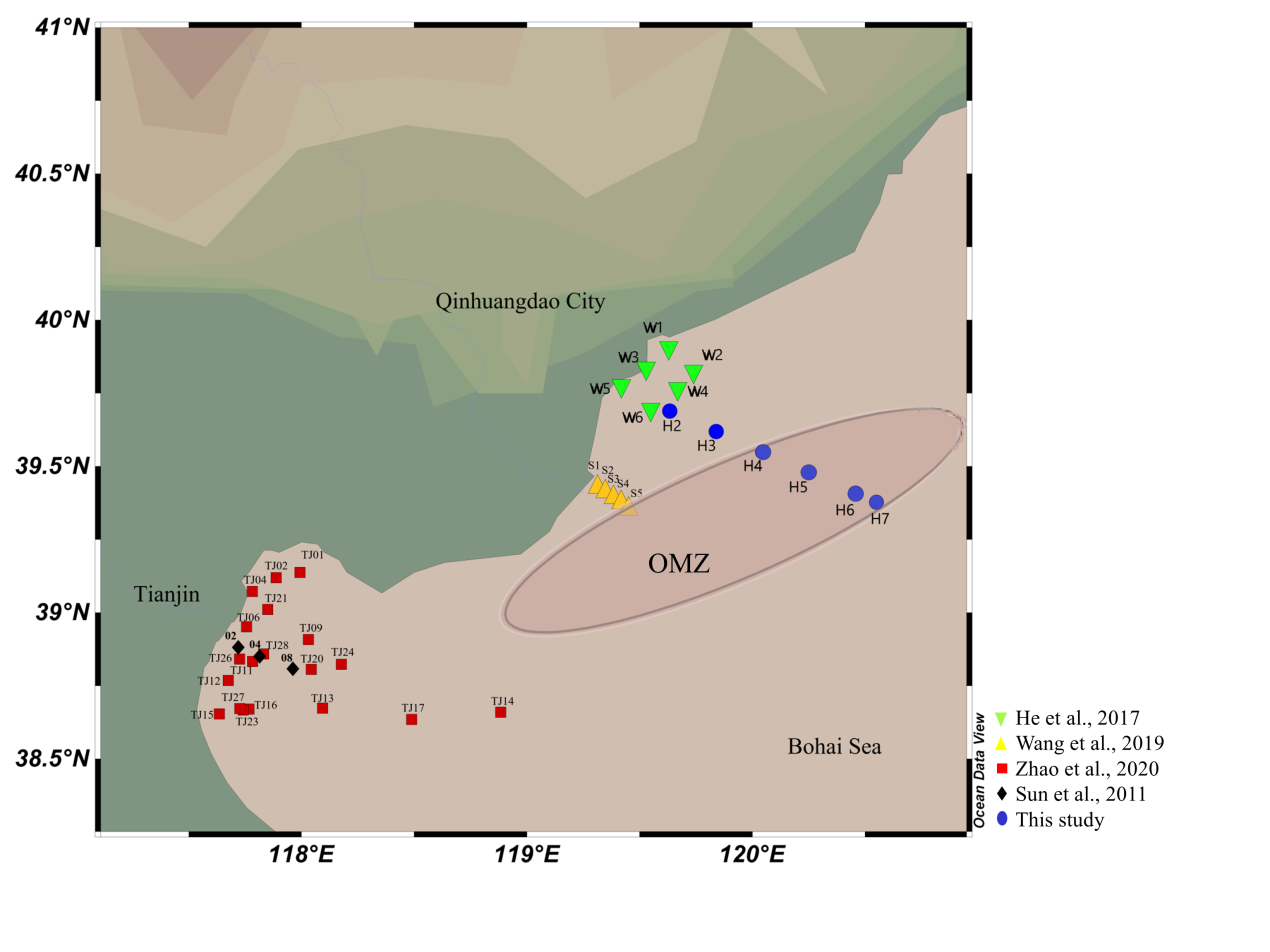
**

**Fig. S3**

Firmicutes

**
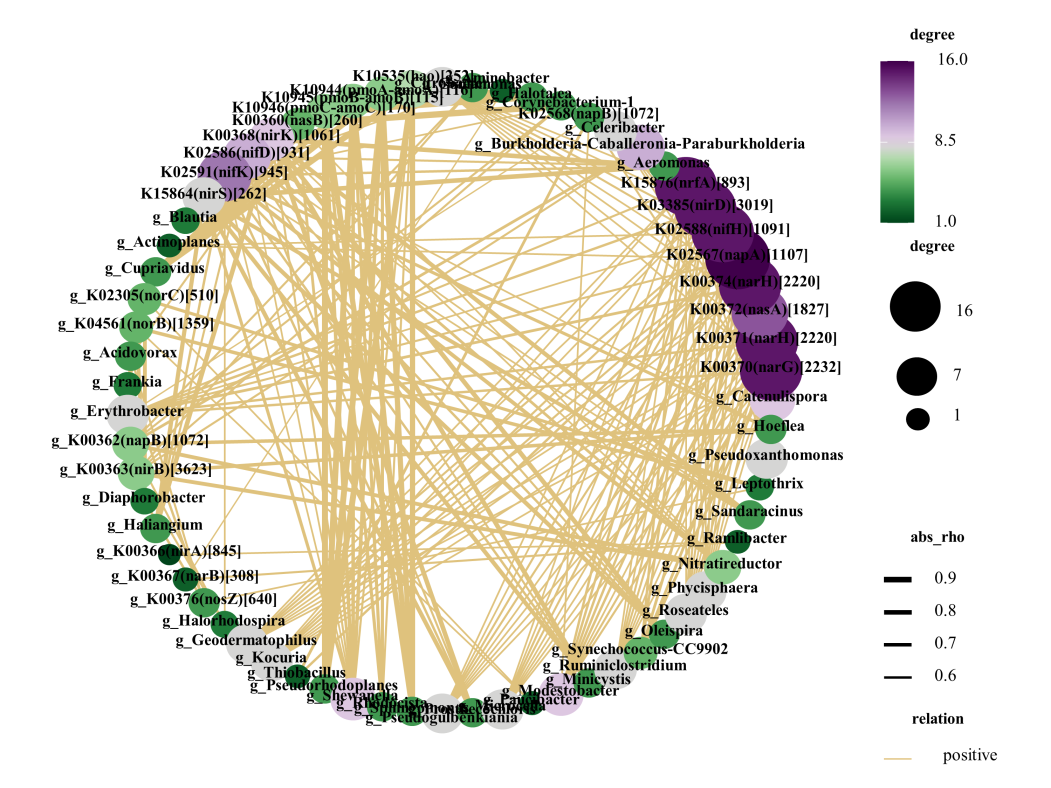
**

**Fig. S4**

Firmicutes

**
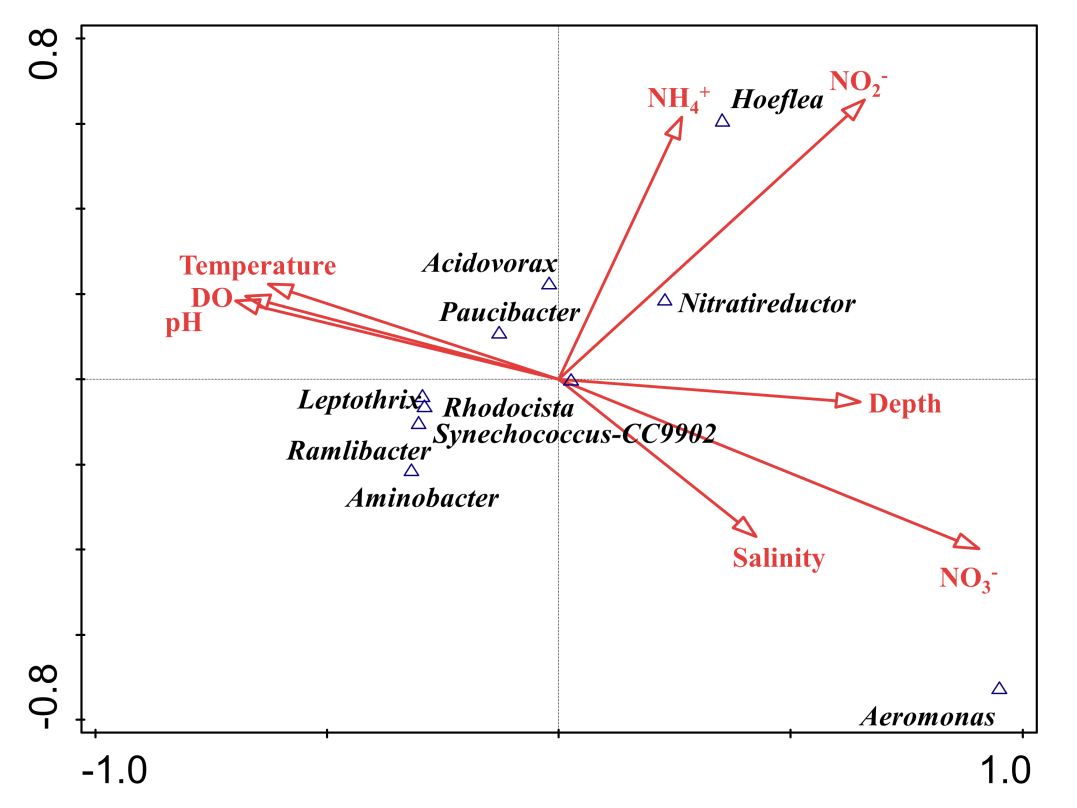
**

**Fig. S5**

Firmicutes

**Table S1 Sample information and environmental parameters of water samples from Bohai low oxygen area.**

| **Sampling date** | **Sites** | **Location** | **Water Depth(m)** | **Sample**  **name** | **Sampling depth (m)** | **Temp  (°C)** | **Salinity** | **Chla (μg/L)** | **DO (mg/L)** | **pH** | **NH_4_ ^+^ （μM）** | **NO_3_ ^-^ （μM）** | **NO₂^-^ （μM）** |
| --- | --- | --- | --- | --- | --- | --- | --- | --- | --- | --- | --- | --- | --- |
| 11, June  2018 | **H2** | 119.634E 39.6883N | 15 | H2S611 | 2 | 18.43 | 32.42 | 0.98 | 8.84 | 8.15 | 0.33 | 0.09 | 0.02 |
|  |  |  |  | H2M611 | 7 | 15.72 | 32.49 | 1.20 | 8.60 | 8.09 | 0.36 | 0.08 | 0.02 |
|  |  |  |  | H2B611 | 13 | 12.26 | 32.59 | 1.61 | 8.24 | 8.02 | 0.65 | 0.22 | 0.05 |
|  | **H3** | 119.840E 39.6183N | 21 | H3S611 | 2 | 17.30 | 32.55 | 1.14 | 8.84 | 8.15 | 0.07 | 0.09 | 0.01 |
|  |  |  |  | H3M611 | 11 | 10.56 | 32.78 | 1.28 | 8.60 | 8.09 | 0.27 | 0.14 | 0.03 |
|  |  |  |  | H3B611 | 19 | 10.36 | 32.63 | 1.11 | 8.44 | 8.03 | 0.49 | 0.19 | 0.04 |
|  | **H4** | 120.046E 39.5483N | 25 | H4S611 | 2 | 19.60 | 32.49 | 0.43 | 8.09 | 8.14 | 0.04 | 0.05 | 0.03 |
|  |  |  |  | H4M611 | 11 | 18.47 | 32.52 | 0.80 | 7.83 | 8.09 | 0.04 | 0.04 | 0.04 |
|  |  |  |  | H4B611 | 23 | 9.48 | 32.76 | 1.18 | 8.90 | 7.99 | 0.69 | 0.53 | 0.09 |
|  | **H5** | 120.252E 39.4783N | 27 | H5S611 | 2 | 19.46 | 32.51 | 0.32 | 8.18 | 8.14 | 0.01 | 0.06 | 0.02 |
|  |  |  |  | H5M611 | 12 | 18.03 | 32.59 | 0.66 | 7.97 | 8.08 | 0.04 | 0.04 | 0.02 |
|  |  |  |  | H5B611 | 25 | 9.38 | 32.73 | 1.63 | 9.16 | 7.99 | 0.47 | 1.12 | 0.13 |
|  | **H6** | 120.458E 39.4083N | 23 | H6S611 | 2 | 19.12 | 32.53 | 0.47 | 8.33 | 8.14 | 0.04 | 0.09 | 0.02 |
|  |  |  |  | H6M611 | 12 | 17.37 | 33.03 | 0.57 | 8.12 | 8.12 | 0.02 | 0.04 | 0.04 |
|  |  |  |  | H6B611 | 20 | 12.52 | 32.64 | 1.89 | 8.52 | 8.04 | 0.15 | 0.14 | 0.04 |
|  | **H7** | 120.551E 39.3765N | 23 | H7S611 | 2 | 19.12 | 32.49 | 0.66 | 8.42 | 8.15 | 0.05 | 0.06 | 0.02 |
|  |  |  |  | H7M611 | 12 | 15.52 | 32.61 | 1.21 | 8.49 | 8.13 | 0.07 | 0.07 | 0.02 |
|  |  |  |  | H7B611 | 20 | 14.72 | 32.56 | 1.09 | 8.39 | 8.05 | 0.37 | 0.17 | 0.05 |
| 19, July  2018 | **H3** | 119.840E 39.6183N | 21 | H3S719 | 2 | 25.64 | 31.48 | 0.95 | 7.73 | 8.15 | 0.15 | 0.08 | 0.02 |
|  |  |  |  | H3M719 | 11 | 18.43 | 32.20 | 2.26 | 7.33 | 8.09 | 0.13 | 0.06 | 0.02 |
|  |  |  |  | H3B719 | 19 | 15.82 | 32.38 | 0.90 | 5.47 | 7.82 | 0.59 | 1.45 | 0.20 |
|  | **H4** | 120.046E 39.5483N | 25 | H4S719 | 2 | 25.08 | 31.58 | 0.79 | 7.62 | 8.18 | 0.14 | 0.05 | 0.03 |
|  |  |  |  | H4M719 | 11 | 16.02 | 31.52 | 2.82 | 7.55 | 8.17 | 0.70 | 0.10 | 0.10 |
|  |  |  |  | H4B719 | 23 | 13.19 | 32.32 | 1.07 | 6.87 | 7.87 | 1.75 | 1.21 | 0.38 |
|  | **H5** | 120.252E 39.4783N | 27 | H5S719 | 2 | 24.12 | 31.63 | 1.09 | 7.59 | 8.20 | 0.15 | 0.03 | 0.03 |
|  |  |  |  | H5M719 | 12 | 13.27 | 32.03 | 1.59 | 6.74 | 7.90 | 0.18 | 1.48 | 0.24 |
|  |  |  |  | H5B719 | 25 | 12.70 | 32.32 | 1.50 | 6.78 | 7.89 | 0.38 | 1.53 | 0.22 |
|  | **H6** | 120.458E 39.4083N | 23 | H6S719 | 2 | 24.50 | 31.00 | 1.61 | 7.72 | 8.23 | 0.14 | 0.02 | 0.04 |
|  |  |  |  | H6M719 | 12 | 14.54 | 32.08 | 2.45 | 6.35 | 7.93 | 0.47 | 1.16 | 0.32 |
|  |  |  |  | H6B719 | 22 | 14.48 | 32.23 | 1.86 | 6.18 | 7.90 | 0.66 | 1.79 | 0.38 |
|  | **H7** | 120.551E 39.3765N | 23 | H7S719 | 2 | 25.08 | 30.39 | 1.47 | 7.74 | 8.27 | 0.24 | 0.04 | 0.03 |
|  |  |  |  | H7M719 | 12 | 17.55 | 31.80 | 1.95 | 6.11 | 7.98 | 0.21 | 1.79 | 0.30 |
|  |  |  |  | H7B719 | 20 | 16.72 | 32.04 | 1.35 | 6.03 | 7.93 | 0.43 | 2.34 | 0.37 |
| 8^th^, August  2018  (Early Aug) | **H3** | 119.840E 39.6183N | 21 | H3S808 | 2 | 26.74 | 31.77 | 2.02 | 7.33 | 8.12 | 0.11 | 0.09 | 0.02 |
|  |  |  |  | H3M808 | 11 | 26.77 | 31.78 | 1.97 | 7.19 | 8.12 | 0.44 | 0.11 | 0.03 |
|  |  |  |  | H3B808 | 19 | 23.11 | 32.01 | 0.48 | 4.85 | 7.85 | 0.71 | 0.63 | 1.07 |
|  | **H4** | 120.046E 39.5483N | 25 | H4S808 | 2 | 26.97 | 31.51 | 1.63 | 7.14 | 8.17 | 0.16 | 0.11 | 0.02 |
|  |  |  |  | H4M808 | 11 | 26.95 | 31.50 | 1.89 | 7.25 | 8.14 | 0.08 | 0.09 | 0.02 |
|  |  |  |  | H4B808 | 23 | 19.02 | 31.97 | 0.26 | 4.66 | 7.81 | 0.04 | 0.53 | 1.98 |
|  | **H5** | 120.252E 39.4783N | 27 | H5S808 | 2 | - | - | 1.36 | - | - | 0.13 | 0.18 | 0.03 |
|  | **H6** | 120.458E 39.4083N | 23 | H6S808 | 2 | 26.93 | 31.45 | 2.11 | 7.42 | 8.11 | 0.04 | 0.12 | 0.03 |
|  |  |  |  | H6M808 | 12 | 22.96 | 30.08 | 4.32 | 5.81 | 7.88 | 0.09 | 0.12 | 0.04 |
|  |  |  |  | H6B808 | 20 | 16.86 | 31.98 | 0.99 | 4.97 | 7.77 | 1.95 | 0.67 | 0.92 |
|  | **H7** | 120.551E 39.3765N | 23 | H7S808 | 2 | 26.96 | 31.32 | 2.11 | 7.97 | 8.14 | 0.06 | 0.19 | 0.02 |
|  |  |  |  | H7M808 | 12 | 19.68 | 31.45 | 4.18 | 4.93 | 7.85 | 0.53 | 0.45 | 0.65 |
|  |  |  |  | H7B808 | 20 | 18.39 | 31.98 | 1.15 | 4.76 | 7.80 | 2.02 | 0.69 | 1.20 |
| 26, August  2018  (Late Aug) | **H3** | 119.840E 39.6183N | 21 | H3S826 | 2 | 26.22 | 30.45 | 1.41 | 7.90 | 8.16 | 0.07 | 0.10 | 0.05 |
|  |  |  |  | H3M826 | 11 | 25.40 | 30.58 | 2.19 | 7.38 | 8.13 | 0.05 | 0.07 | 0.08 |
|  |  |  |  | H3B826 | 19 | 25.02 | 30.55 | 1.65 | 6.40 | 8.06 | 0.13 | 0.83 | 1.04 |
|  | **H4** | 120.046E 39.5483N | 25 | H4S826 | 2 | 25.87 | 30.79 | 1.16 | 7.90 | 8.19 | 0.07 | 0.12 | 0.05 |
|  |  |  |  | H4M826 | 11 | 25.46 | 30.87 | 1.79 | 7.66 | 8.16 | 0.06 | 0.11 | 0.09 |
|  |  |  |  | H4B826 | 23 | 23.45 | 31.26 | 1.04 | 4.72 | 7.93 | 0.06 | 0.16 | 0.05 |
|  | **H5** | 120.252E 39.4783N | 27 | H5S826 | 2 | 25.56 | 31.94 | 0.89 | 7.69 | 8.17 | 0.06 | 0.10 | 0.02 |
|  |  |  |  | H5M826 | 12 | 25.49 | 30.88 | 1.73 | 7.73 | 8.16 | 0.05 | 0.07 | 0.03 |
|  |  |  |  | H5B826 | 25 | 21.97 | 31.66 | 0.90 | 4.21 | 7.88 | 0.05 | 5.62 | 0.41 |
|  | **H6** | 120.458E 39.4083N | 23 | H6S826 | 2 | 24.67 | 31.18 | 0.65 | 7.40 | 8.14 | 0.03 | 0.09 | 0.02 |
|  |  |  |  | H6M826 | 12 | 23.31 | 31.21 | 2.43 | 6.25 | 8.05 | 0.07 | 1.05 | 0.42 |
|  |  |  |  | H6B826 | 20 | 22.71 | 31.29 | 1.70 | 5.54 | 7.99 | 0.07 | 0.13 | 0.02 |
|  | **H7** | 120.551E 39.3765N | 23 | H7S826 | 2 | 24.39 | 31.28 | 0.77 | 7.31 | 8.11 | 0.06 | 0.09 | 0.02 |
|  |  |  |  | H7M826 | 12 | 23.46 | 31.38 | 2.29 | 6.90 | 8.06 | 0.05 | 1.24 | 0.41 |
|  |  |  |  | H7M2826 | 16 | 22.54 | 31.37 | 2.70 | 5.24 | 7.95 | 0.05 | 0.92 | 0.40 |
|  |  |  |  | H7B826 | 20 | 22.17 | 31.55 | 1.79 | 5.18 | 7.94 | 0.07 | 2.20 | 0.48 |

**Table S2 Environmental parameters driving the distribution of each sample group.**

| Sample Group | Parameters | Explains % | Contribution % | pseudo-F | *p* |
| --- | --- | --- | --- | --- | --- |
| All samples | Depth | 17.80 | 36.90 | 12.80 | 0.002 |
|  | Salinity | 11.50 | 23.80 | 9.40 | 0.002 |
|  | Chl*a* | 6.30 | 13.20 | 5.60 | 0.004 |
|  | NO_2_^-^ | 4.20 | 8.60 | 3.90 | 0.012 |
|  | pH | 2.80 | 5.90 | 2.80 | 0.042 |
| August samples | DO | 26.50 | 38.30 | 9.40 | 0.002 |
|  | NO_2_^-^ | 16.00 | 23.10 | 6.90 | 0.002 |
|  | Chl*a* | 8.20 | 11.90 | 4.00 | 0.020 |
| Bottom water samples | NO_2_^-^ | 17.10 | 23.30 | 4.00 | 0.032 |

Items in red reached an extremely significant level (*p*<0.01); items in blue reached a significant level (*p*<0.05).

**Table S3 Correlation of nosZ-denitrifiers and functional genes related to denitrification**

| **Correlation**  ***p* value** | **K00370 (narG)[2232]** | **K00371 (narH)[2220]** | **K00374 (narH)[2220]** | **K02567 (napA)[1107]** | **K00362 (napB)[1072]** | **K00368 (nirK)[1061]** | **K15864 (nirS)[262]** | **K04561 (norB)[1359]** | **K02305 (norC)[510]** | **K00376 (nosZ)[640]** |
| --- | --- | --- | --- | --- | --- | --- | --- | --- | --- | --- |
| ***Catenulispora*** | 0.01 | 0.01 | 0.01 | 0.01 | / | / | / | / | / | / |
| ***Kocuria*** | 0.04 | 0.04 | 0.04 | 0.03 | / | / | / | / | / | / |
| ***Burkholderia-Caballeronia-Paraburkholderia*** | 0.03 | 0.03 | 0.03 | 0.05 | / | / | / | / | / | / |
| ***Celeribacter*** | 0.04 | 0.04 | 0.04 | 0.03 | / | / | / | / | / | / |
| ***Citrobacter*** | 0.04 | 0.04 | 0.04 | 0.03 | / | / | / | / | / | / |
| ***Pseudogulbenkiania*** | 0.04 | 0.04 | 0.04 | 0.03 | / | / | / | / | / | / |
| ***Microcella*** | 0.04 | 0.04 | 0.04 | 0.03 | / | / | / | / | / | / |
| ***Modestobacter*** | 0.02 | 0.02 | 0.02 | 0.02 | / | / | / | / | / | / |
| ***Ruminiclostridium*** | 0.04 | 0.04 | 0.04 | 0.03 | / | / | / | / | / | / |
| ***Synechococcus-CC9902*** | 0.01 | 0.01 | 0.01 | / | / | / | / | / | / | / |
| ***Erythrobacter*** | 0.01 | 0.01 | 0.01 | 0.02 | / | / | / | / | / | / |
| ***Roseateles*** | 0.04 | 0.04 | 0.04 | 0.03 | / | / | / | / | / | / |
| ***Phycisphaera*** | 0.04 | 0.04 | 0.04 | 0.03 | / | / | / | / | / | / |
| ***Geodermatophilus*** | 0.04 | 0.04 | 0.04 | 0.03 | / | / | / | / | / | / |
| ***seudoxanthomo/s*** | 0.04 | 0.04 | 0.04 | 0.03 | / | / | / | / | / | / |
| ***Thiobacillus*** | / | / | / | 0.02 | / | / | / | / | / | / |
| ***Actinoplanes*** | / | / | / | 0.04 | / | / | / | / | / | / |
| ***Burkholderia-Caballeronia-Paraburkholderia*** | / | / | / | / | 0.01 | / | / | / | / | / |
| ***Corynebacterium-1*** | / | / | / | / | 0.02 | / | / | / | / | / |
| ***Comamo/s*** | / | / | / | / | 0.02 | / | / | / | / | / |
| ***Nitratireductor*** | / | / | / | / | 0.00 | / | / | / | / | / |
| ***Halorhodospira*** | / | / | / | / | 0.02 | / | / | / | / | / |
| ***oeflea*** | / | / | / | / | 0.00 | / | / | / | / | / |
| ***Aeromo/s*** | / | / | / | / | / | 0.00 | 0.00 | / | / | / |
| ***Shewanella*** | / | / | / | / | / | 0.02 | 0.00 | / | / | / |
| ***Blautia*** | / | / | / | / | / | 0.00 | 0.01 | / | / | / |
| ***Prosthecochloris*** | / | / | / | / | / | 0.00 | 0.00 | / | / | / |
| ***Minicystis*** | / | / | / | / | / | 0.00 | 0.00 | / | / | / |
| ***Oleispira*** | / | / | / | / | / | 0.00 | 0.00 | / | / | / |
| ***Nitratireductor*** | / | / | / | / | / | 0.04 | / | / | / | / |
| ***Haliangium*** | / | / | / | / | / | 0.00 | 0.00 | / | / | / |
| ***Sandaracinus*** | / | / | / | / | / | 0.00 | 0.00 | / | / | / |
| ***Paucibacter*** | / | / | / | / | / | / | / | 0.04 | 0.04 | / |
| ***Acidovorax*** | / | / | / | / | / | / | / | 0.01 | 0.01 | 0.03 |
| ***Frankia*** | / | / | / | / | / | / | / | 0.00 | 0.00 | 0.02 |
| ***Synechococcus-CC9902*** | / | / | / | / | / | / | / | 0.03 | 0.02 | 0.01 |
| ***Diaphorobacter*** | / | / | / | / | / | / | / | 0.00 | 0.00 | 0.02 |
| ***Leptothrix*** | / | / | / | / | / | / | / | 0.00 | 0.00 | 0.03 |
| ***Prochlorococcus-MIT9313*** | / | / | / | / | / | / | / | / | / | 0.04 |

“/” nosZ-denitrifiers and functional genes related to denitrification have no significant correlation.

**Table S4 Pearson correlations and significance between nutrients and bacteria composition.**

|  | **NO₂^-^（μM）** | | **NH_4_ ^+^（μM）** | | | **NO_3_ ^-^（μM）** | | | |  |
| --- | --- | --- | --- | --- | --- | --- | --- | --- | --- | --- |
|  | ***r*** | *p* | ***r*** | | ***p*** | | *r* | | ***p*** | |
| Cyanobacteria | -0.429^**^ | 0.010 | -0.438^**^ | 0.000 | | -0.352^**^ | | 0.005 | |  |
| Proteobacteria | 0.503^**^ | 0.000 | 0.474^**^ | 0.000 | | 0.042^**^ | | 0.001 | |  |
| Verrucomicrobia | -0.310^*^ | 0.014 | -0.035 | 0.790 | | -0.232 | | 0.070 | |  |
| Actinobacteria | 0.436^**^ | 0.000 | 0.144 | 0.264 | | 0.404^**^ | | 0.001 | |  |
| Firmicutes | -0.119 | 0.356 | 0.027 | 0.835 | | -0.113 | | 0.384 | |  |
| Bacteroidetes | -0.041 | 0.750 | 0.036 | 0.779 | | -0.109 | | 0.398 | |  |
| Planctomycetes | 0.256^*^ | 0.044 | -0.008 | 0.953 | | 0.180 | | 0.163 | |  |

** Correlation is significant at *p*≤0.01.

* Correlation is significant at *p*≤ 0.05.
